# Photo-induced H_2_ production from a CH_3_OH-H_2_O solution at insulator surface

**DOI:** 10.1038/srep13475

**Published:** 2015-08-28

**Authors:** Rengui Li, Xiuli Wang, Shaoqing Jin, Xin Zhou, Zhaochi Feng, Zheng Li, Jingying Shi, Qiao Zhang, Can Li

**Affiliations:** 1 State Key Laboratory of Catalysis, Dalian Institute of Chemical Physics, Chinese Academy of Sciences, Dalian National Laboratory for Clean Energy, The Collaborative Innovation Center of Chemistry for Energy Materials (iChEM), Zhongshan Road 457, Dalian, 116023, China; 2 University of Chinese Academy of Sciences, Beijing, 100049, China

## Abstract

In a conventional photocatalytic or photochemical process, either a photocatalyst or a molecule is excited by irradiation light that has energy greater than the forbidden band (i.e., the band gap) of the semiconductor or the transition energy of an excited state of the molecule, respectively, for a reaction to occur. However, in this work, we found that a considerable amount of H_2_ can be generated from a CH_3_OH-H_2_O solution at a quartz surface using light with energy far outside the electronic absorbance range of the CH_3_OH-H_2_O solution; this process should not occur in principle via either conventional photocatalysis or a photochemical process. The H_2_ production was further confirmed using 266 nm and 355 nm lasers as light sources. Our work demonstrates that photo-induced H_2_ production can occur on insulator surfaces (e.g., quartz), which were commonly believed to be inert, and will shed light on the surface nature of insulators.

According to conventional photocatalysis theory, when the energy of incident light is larger than the band gap of a semiconductor-based photocatalyst (E_λ_ ≥ E_g_), electrons and holes can be generated in the conduction and valance bands for reduction and oxidation reactions, respectively^1^,^2^,^3^,^4^,^5^. Meanwhile, a photochemical reaction is a chemical process initiated by the absorption of photo energy. The absorption of light by molecules leads to electronically excited states in molecules. Thus, molecules must be excited from the HOMO to the LUMO by photons with sufficient energy^6^,^7^,^8^,^9^. Therefore, both photocatalytic and photochemical processes require sufficient energy, that is, energy greater than the forbidden band (i.e., band gap) or the transition energy of an excited state.

The CH_3_OH molecule is frequently used as a hole scavenger (sacrificial reagent) in photocatalytic H_2_ production, and this molecule can trap the photoexcited holes of a semiconductor so that photogenerated electrons participate in proton reduction^3^,^4^,^5^,^10^,^11^. It was generally believed that CH_3_OH itself cannot contribute to H_2_ production because it does not absorb any light from commonly used light sources. Almost all the reported photocatalysts are semiconductor-based materials with a suitable band structure, but none of the insulators can be used for photocatalytic hydrogen production because their band gaps are too large to be excited by the usual UV and visible light sources.

However, in this work, we found that a considerable amount of H_2_ can be generated from a CH_3_OH-H_2_O solution at an insulator surface using light far outside the electronic absorption range of CH_3_OH; this process does not occur via either conventional photocatalysis or a photochemical process. Incident light with a wavelength up to 400 nm can even induce H_2_ production from the CH_3_OH-H_2_O solution. When insulator oxide (SiO_2_ or Al_2_O_3_) particles onto which Pt has been deposited were added to the reaction solution, the H_2_ production was strongly enhanced. Photoluminescence and EPR data suggested that electrons at the surface states of the insulator are possibly to be excited from the valence band of insulators (e.g., quartz, SiO_2_ or Al_2_O_3_), and this process may be responsible for the H_2_ production via electron-proton coupling with the CH_3_OH-H_2_O solution.

## Results

### Photo-induced H_2_ production using a CH_3_OH-H_2_O solution

**Figure 1:**
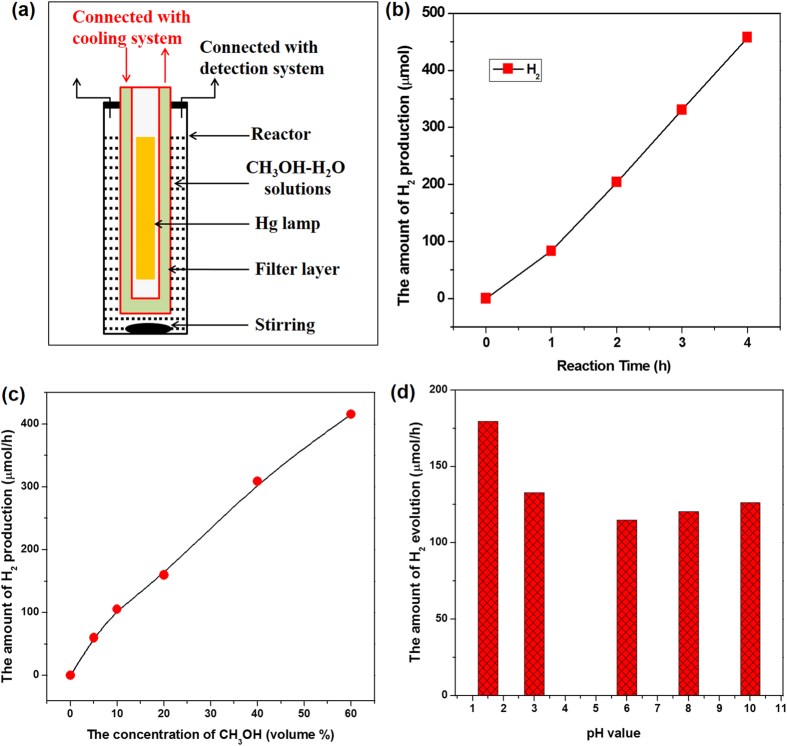
(**a**) The configuration of reactor used in the experiment; (**b**) photo-induced H_2_ production from a CH_3_OH-H_2_O solution without photocatalyst under the light irradiation; (**c**) The concentration dependence of the H_2_ production from the CH_3_OH-H_2_O solution; (**d**) The pH dependence of the H_2_ production from the CH_3_OH-H_2_O solution, the pH value of solution was adjusted with H_2_SO_4_ or NaOH solution (1.0 mol/L). Reaction condition: 500 mL CH_3_OH-H_2_O solution, the concentration of CH_3_OH was 10% in volume in (**b**,**d**); 450 W high-pressure Hg lamp; Pure CH_3_OH (>99.99%) and pure water (18 MΩ H_2_O, obtained from a Milli-Q water purification system) was used in the experiment.

The experiment was conducted using the typical widely-used setup for evaluating photocatalytic H_2_ production but without the addition of any photocatalyst. A high-pressure Hg lamp was employed as the light source; this type of source has commonly been used to evaluate semiconductor-based photocatalysts (Figure S1). The Hg lamp is located inside the reactor so that the light can pass through the reactor wall to the CH_3_OH-H_2_O solution (Fig. 1a and Figure S2). To obtain the required range of light source, different light-absorbing solutions are filled in the filter layer (quartz-made) to filter the light by absorbing a specific range of light. After the reaction, the produced gas fills a glass-made closed system and is connected to a gas chromatography (GC) apparatus for analysis.

Figure 1b shows the time course of H_2_ production from a CH_3_OH-H_2_O solution under light irradiation. Surprisingly, H_2_ was detected even without the addition of any conventional photocatalyst. The amount of H_2_ increased linearly with irradiation time, and the H_2_ production rate was approximately 100 μmol/h; this rate is almost the same as the rate that has been reported for methanol reformation on some photocatalysts^3^. We then performed a long-time reaction, and the H_2_ production activity could be well maintained for irradiation lasting longer than 24 hours.

Several experiments were then performed to confirm the extraordinary H_2_ production. First, the concentration dependence of the H_2_ production using the CH_3_OH-H_2_O solution was measured. Figure 1c shows that the H_2_ production increased almost linearly with the concentration of the CH_3_OH solution, indicating that the H_2_ is most likely produced from CH_3_OH. The H_2_ production rate could exceed 400 μmol/h when the concentration of CH_3_OH was increased to 60% of the volume; this rate is approximately 4 times higher than the rate shown in Fig. 1b. Second, the pH dependence of the H_2_ production using the CH_3_OH-H_2_O solution was measured, and the H_2_ production rate was maintained for pH values between 3.0 and 10.0 but slightly increased when the pH value was lower than 1.5 (Fig. 1d). Third, we also introduced some other organic reagents (e.g., ethanol, isopropyl alcohol and lactic acid) to replace CH_3_OH. As shown in Table S1, a considerable amount of H_2_ was obtained for all these cases, which implies the generality of this extraordinary H_2_ production. All these results clearly indicate that H_2_ can indeed be generated from a CH_3_OH-H_2_O solution even without the addition of any conventional photocatalyst.

### Photo-induced H_2_ production under different wavelengths

To verify which region of light can induce the H_2_ production from a CH_3_OH-H_2_O solution, we used different solutions to filter the short-wavelength light at wavelengths approximately below 240 nm, 340 nm, or 400 nm (Figure S3). At the given wavelength range of the light source, photo-induced H_2_ production from a CH_3_OH-H_2_O solution was performed. As shown in Table 1, a small amount of H_2_ can even be detected when light shorter than 400 nm was blocked (entry 2). However, the H_2_ production rate could increase to approximately 2% of that of the full spectrum when the irradiation light was longer than 340 nm (entry 3). This value could increase to 10% when the irradiation light was longer than 300 nm (Figure S4 and entry 4). The H_2_ production rate was further increased to a level comparable to that of the full spectrum when the irradiation light was longer than 240 nm because there is no obvious light peak shorter than 240 nm for a Hg lamp (entries 1 and 5).

To confirm the role of the light wavelength in H_2_ production, a Xe lamp (300 W) was used to replace the Hg lamp as the light source. The emission spectrum of the Xe lamp shows a continuous spectrum from 300 nm to the visible region especially without any peaks at wavelength shorter than 300 nm (Figure S5). The result shows that only a trace of H_2_ is observed after a reaction for 12 hours, demonstrating that the light above 300 nm has a negligible contribution to the H_2_ production. The difference between the Xe and the Hg lamp is mainly in the UV range below 300 nm. Comparing the H_2_ production of the two different light sources indicates that H_2_ is mainly produced from the CH_3_OH-H_2_O solution using light between 240 and 300 nm. All the results clearly indicate that H_2_ can be produced from a CH_3_OH-H_2_O solution even without conventional photocatalyst. To further investigate the origin and mechanism of the H_2_ production, several possible factors have to be considered, and controlled experiments were conducted as follows.

## Discussion

A mechanical-to-chemical energy conversion process was able to produce H_2_ and O_2_ in a photocatalytic evaluation system when mechanical stirring was used in the presence of some metal oxides^12^,^13^,^14^,^15^,^16^,^17^,^18^,^19^. A blank experiment with only mechanical stirring was first investigated under the same condition as for the photo-induced H_2_ production. The result revealed that no H_2_ was detected even after a test for more than 24 hours, so a contribution from the mechanical-to-chemical energy process could be ruled out using this experiment.

**Figure 2:**
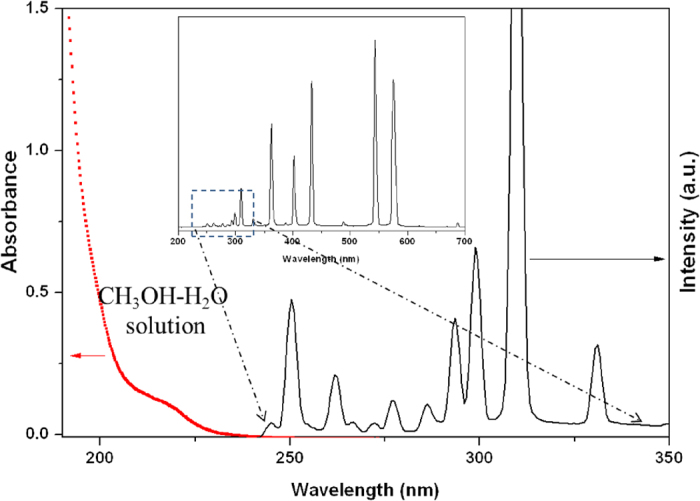
The comparison of the absorption of the CH_3_OH-H_2_O solution and the photo-emission of Hg lamp. The emission spectrum of Hg lamp was characterized by a commercial spectral radiometer (AvaSolar).

Because no photocatalyst was used in the experiment, it seemed unlikely that the H_2_ production could be attributed to a traditional photochemical process, in which the CH_3_OH-H_2_O solution would absorb light to make this reaction happen. It has been reported that CH_3_OH in gas phase can decompose through a photochemical process involving light shorter than 200 nm^20^,^21^,^22^,^23^ because the absorption edge of gaseous CH_3_OH molecules is below 200 nm, namely, in the deep UV region^24^,^25^,^26^. Figure 2 shows that the absorption edge of the CH_3_OH-H_2_O solution is located at approximately 230 nm, but the emission spectrum of the Hg lamp source shows no peaks shorter than 240 nm (note, the quartz reactor can completely block light below 200 nm, while the glass reactor can completely block light below 300 nm even when a Hg light source is used), which means that there is no overlap between the absorption spectrum of the CH_3_OH-H_2_O solution and the emission spectrum of the traditional light source (e.g., Hg lamp or Xe lamp). To confirm this result, we also conducted the experiment in pure H_2_O. In this case, the absorption of H_2_O is much shorter than the emission spectrum of Hg lamp, indicating that it is impossible to excite H_2_O molecular by Hg lamp (Figure S6a). However, we can achieve both H_2_ and O_2_ in pure H_2_O although the activity is low enough, which indicating that overall water splitting can take place under the light irradiation (Figure S6b and S6c). Furthermore, it was found that the water splitting performance can be improved by adding quartz particles in the solution. So the above results indicate that the H_2_ is produced not by a conventional photochemical process.

**Figure 3:**
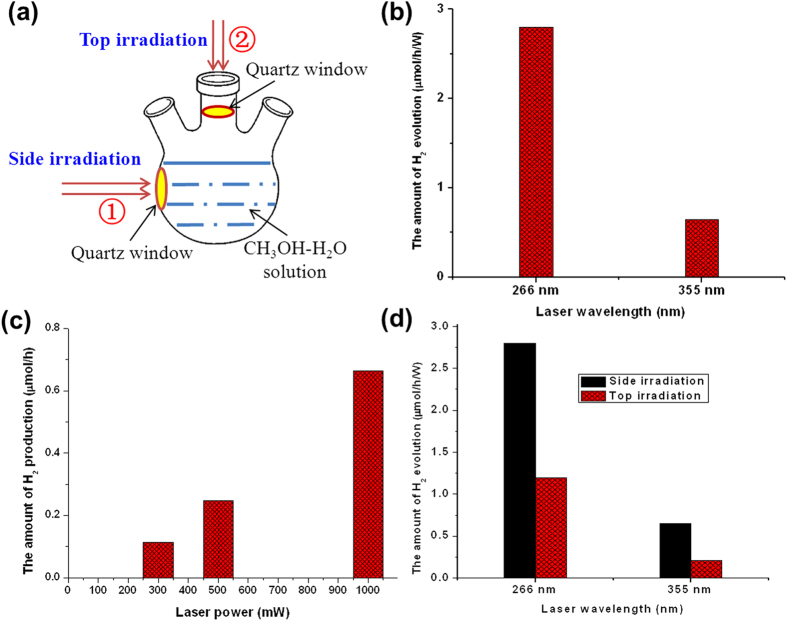
Photo-induced H_2_ production from a CH_3_OH-H_2_O solution using lasers as light source. (**a**) The reactor scheme of the two types of irradiation from different directions, side irradiation and top irradiation; (**b**) Photo-induced H_2_ production under the irradiation of two different lasers (266 nm and 355 nm); (**c**) The comparison of photo-induced H_2_ production via two types of irradiation; (**d**) Photo-induced H_2_ production under irradiation by the 355 nm laser at different laser powers. Reaction condition: 100 mL CH_3_OH-H_2_O solution (50% CH_3_OH), irradiation time: 2 h. The system was first vacuumed and saturated with Ar, and then irradiated with 266 nm and 355 nm lasers. The laser at 355 nm of a Nd:YAG laser was used as exciting source and the laser at 266 nm comes from the double-frequency of a DPSS 532 Model 200 532 nm laser. The produced H_2_ was sampled with injector and analyzed with GC.

To avoid the possibility that any weak emissions in the deep UV region below 240 nm would attribute to the H_2_ production from CH_3_OH-H_2_O solution, two lasers with a single frequency of either 355 nm or 266 nm were used as light sources (the reactor scheme is shown in Fig. 3a). Figure 3b shows the H_2_ production under the irradiation of two different lasers. Interestingly, we found that H_2_ is indeed produced under the irradiation of both the 266 nm and 355 nm lasers. Notably, the laser wavelength of 355 nm is more than 100 nm greater than the absorption edge of the CH_3_OH-H_2_O solution, that is, there is no electronic absorption by the CH_3_OH-H_2_O solution in this range. In addition, the H_2_ production rate under 266 nm irradiation is 4 times that under 355 nm irradiation when normalized by the laser power. This result unambiguously proves that irradiation light outside the electronic absorption range of a CH_3_OH-H_2_O solution can indeed induce H_2_ production. Figure 3c shows the photo-induced H_2_ production under irradiation by the 355 nm laser at different laser powers (300 mW, 500 mW and 1000 mW). As the laser power increased, the H_2_ production increased linearly. We also conducted the experiment using Pyrex window under the irradiation of 266 nm and 355 nm lasers from side (Table S3). It can be found that the H_2_ production is greatly decreased after replacing the quartz-window to Pyrex-window both for 266 nm and 355 nm lasers. Besides, we also performed the H_2_ production experiment using Pyrex-made filter layer under the irradiation of Hg lamp, in this case, the lamp spectrum shorter than 300 nm is filtered, the amount of H_2_ production is remarkably decreased to 10% of that for quartz-made filter layer (from 83 μmol/h to 8.0 μmol/h).

To demonstrate the effect of the interface between the quartz window and the CH_3_OH-H_2_O solution on H_2_ production, two types of irradiation from different directions (side irradiation and top irradiation) using the 266 and 355 nm lasers were applied (Fig. 3d). The result shows that the H_2_ production using the side irradiation was much higher than that of the top irradiation. All the above results indicate that the interface between the quartz surface and the solution plays an important role in photo-induced H_2_ production from the CH_3_OH-H_2_O solution. Because the CH_3_OH-H_2_O solution did not show any electronic absorption at either 266 nm or 355 nm, we can conclude that the H_2_ production does not originate from the conventional photochemical process of CH_3_OH decomposition.

**Figure 4:**
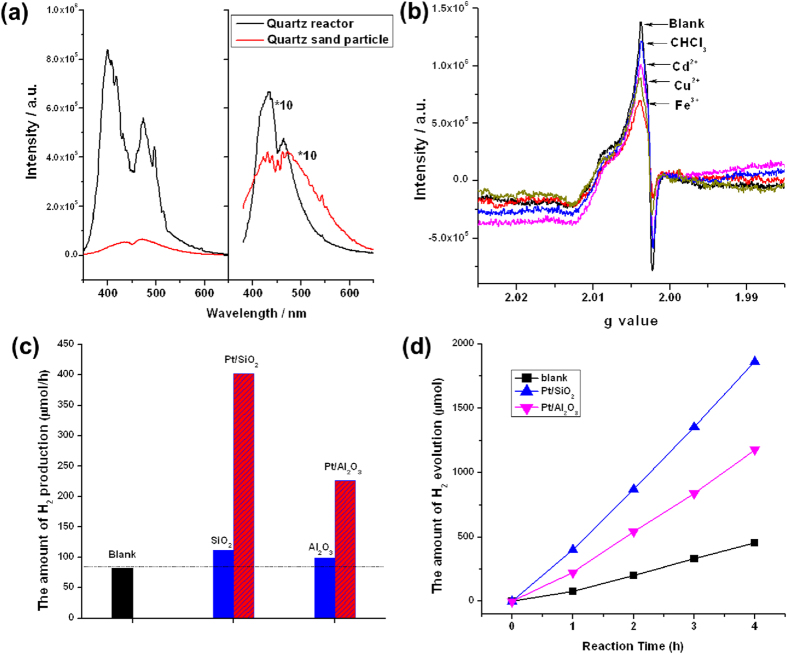
(**a**) Photoluminescence spectra of quartz reactor and quartz sand powders under excitation by the 266 nm and 325 nm lasers. (**b**) EPR spectra of the quartz sand particles with or without treatment by different electron scavengers. (**c**) Photo-induced H_2_ production with the addition of insulator particles (SiO_2_ or Al_2_O_3_) into the CH_3_OH-H_2_O solution. (**d**) The time curve of photo-induced H_2_ production in (**c**). Reaction condition: 5.0 g insulator particles were added in the solution, 0.05 wt% Pt was deposited by *in-situ* photo-deposition method at the initial stage of the reaction, 500 mL CH_3_OH-H_2_O solution (10% CH_3_OH), 450 W Hg lamp was used as light source, inner irradiation type.

It turns out that the H_2_ is most likely produced at the interface between the quartz surface and the CH_3_OH-H_2_O solution. The quartz-made filter layer are connected directly with CH_3_OH-H_2_O solution and has a large interface with solution. The quartz is made of SiO_2_ that is calcined at high temperatures (usually over 700 °C). Some defect sites is possibly to be generated on the quartz surface after calcination at high temperatures, and these defect sites may act as electron acceptors, making excitation from the valance band of quartz to these states possible. Similarly, the existence of this type of defect state in other metal oxides (e.g., Fe_2_O_3_) has been demonstrated in previous work^27^,^28^. To demonstrate this possibility, photoluminescence spectra were measured using the quartz-made filter layer and quartz sand particles under excitation by 266 nm and 325 nm lasers. As shown in Fig. 4a, the quartz-made filter layer exhibited an emission range from 350 nm (3.54 eV) to 600 nm (2.06 eV), indicating that there are electrons possibly present in the defect states of quartz, which could be excited by both the 266 nm and 325 nm lasers; this finding further confirmed that the electrons in these defect states can be excited by a Hg lamp.

To verify the existence of the defect states deduced from the PL spectra, we used electron paramagnetic resonance (EPR) to characterize the quartz sand particles. As shown in Fig. 4b, the EPR signals with a g value between 2.00 and 2.01 could be ascribed to the electrons in the defect states in quartz according to references^29^,^30^ and could thus possibly correspond to the electrons in the defect states observed in the PL spectra. Because the surface defect states can easily be quenched by electron scavengers, we performed EPR characterization after treatment with different electron scavengers. Some metal ions (e.g., Fe^3+^ and Cu^2+^) are generally recognized as strong electron acceptors in photocatalysis; meanwhile, Cd^2+^ and CHCl_3_ are electron scavengers that are used to quench photoexcited electrons^27^. The result showed that the intensity of the EPR signals decreased after the treatment with these electron scavengers, suggesting that some electrons in these defect states could really be quenched by electrons scavengers. We also collected EPR signals under UV light irradiation, and the result showed that the EPR signals can be recovered somewhat for all the electron scavenger-treated systems, indicating that amount of electrons in the defects states could be increased by UV light irradiation (Figure S7). Figure S8 clearly shows the net increase in the EPR signals after the light irradiation, which can be attributed to the photo-induced electrons in the defect states. Electron scavengers were also introduced to investigate the influence on the photo-induced H_2_ production. It was found that the H_2_ production rate could really be suppressed when some electron scavengers were added to the reaction system, which is in good agreement with the EPR results of Fig. 4b (Table S2). These results indicate that the introduction of electron scavengers can partly quench the photoexcited electrons at the quartz surface. The above results led us to the conclusion that the electrons in the defect states detected by the PL and EPR spectra are at least partly attributed to the surface states of the quartz, which would be the origin of the photo-induced H_2_ production from the CH_3_OH-H_2_O solution.

Moreover, we intentionally introduced some commercial quartz sand particles into the CH_3_OH-H_2_O solution to increase the contact interfaces between the solution and quartz. The H_2_ production was enhanced by the addition of the quartz sand particles, and the activity was able to increase up to 1.4 times (Figure S9). Furthermore, the H_2_ production rate can be increased by more than 4 times after a Pt cocatalyst was deposited on these quartz sand particles. It is well known that Pt is an efficient catalyst for proton reduction, so electrons could be quickly accepted when a Pt cocatalyst is deposited on quartz sand particles. The utilization efficiency of electrons could therefore be greatly improved, which led to a significant improvement in the H_2_ production (Fig. 4c). As shown in Fig. 4d, the H_2_ production for these cases increased linearly with irradiation time. The result indeed shows that the Pt particles could accept the photogenerated electrons from the surface states of the insulator. A similar result was obtained when Al_2_O_3_ particles were added to the reactor, suggesting that this phenomenon could be general for insulators. It was reported that the band gaps of Al_2_O_3_ and SiO_2_ are 8.8 eV and 9.0 eV, respectively^31^,^32^, which means that both materials could only be excited by light with a wavelength shorter than 140 nm. Calculation of the total density of states (DOS) of SiO_2_ indicates that the valence band maximum is mainly composed of the by O 2p orbital, which is similar to the situation for some common oxide semiconductors (Figure S10). It is reasonable to introduce some widely known metal oxides semiconductors (e.g., TiO_2_ or ZnO) as references to estimate the energy level of the surface states^33^. In contrast to these metal oxides, quartz (SiO_2_) has a valence band maximum that is estimated to be located between 1.92 and 2.91 eV (Figure S11). The photoluminescence bands excited by 266 and 325 nm lasers suggest that the energy levels of the surface states for quartz are at least 2.06 eV to 3.54 eV above the valence band. Thus, we can speculate that most of the excited electrons at these surface states could have sufficient energy for H_2_ production through proton-electron coupling.

The result implies that the H_2_ production occurs at the interface between insulator surfaces (e.g., quartz) and the CH_3_OH-H_2_O solution. Except for the H_2_ production, we analyzed the products in the liquid phase, and HCHO was detected after the photo-induced H_2_ production reaction. Both H_2_ and HCHO were detected in stoichiometric ratio and increased with the reaction time. Thus, the entire reaction can be summarized in the following equations (1–3).


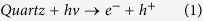



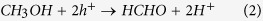



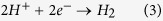


Based on the above results and discussion, the surface states of insulators located between the band gap may act as electron acceptors, which may contribute to the H_2_ production in the CH_3_OH-H_2_O solution even in the absence of a conventional photocatalyst. This possible mechanism is reasonable as the similar mechanism has been reported on a UV-light-responsible photocatalyst, Nb_2_O_5_ (Eg = 3.2 eV), which can be excited by visible light when a donor level consisting of a N 2p orbital was introduced^34^,^35^,^36^.

It should be noted that the CH_3_OH molecule is frequently used as a sacrificial reagent for photocatalytic H_2_ production, as reported in a vast amount of literature. However, our result indicates that H_2_ could be produced from a CH_3_OH-H_2_O solution in the absence of a photocatalyst and under UV light irradiation (λ: 240–400 nm). This finding means that a certain amount of H_2_ may originate from the blank experiment instead of the photocatalytic process on the photocatalyst. In order to investigate how much difference of H_2_ production performance with or without photocatalyst, we introduced commercial Degussa P25 as a benchmark photocatalyst (Table S4). It was found that the H_2_ production on P25 photocatalyst (Pt as cocatalyst) could be enhanced more than 30 times (from 83.1 to 2795.3 μmol/h) than the blank one. It indicates that the effect H_2_ production from blank experiment is only 3% contribution compared with P25 photocatalyst, which seems that the H_2_ production from the blank experiment could be always neglected for some high activity photocatalyst systems. However, it may lead to a wrong conclusion when the activity of photocatalyst is low enough. Unfortunately, this possibility has been ignored in most of the photocatalytic H_2_ production work reported in the literature when a Hg lamp is used as a light source.

In summary, we found that H_2_ can be generated from a CH_3_OH-H_2_O aqueous solution at an insulator (e.g., quartz, SiO_2_ or Al_2_O_3_) surface using light far outside the electronic absorption of the CH_3_OH-H_2_O solution, and this process does not occur via either conventional photocatalysis or a photochemical process. This work shows the fact that photo-induced H_2_ production can take place on insulator surfaces (e.g., quartz), which are commonly believed to be inert, and will shed light on the surface nature of insulators.

## Methods

### The evaluation of H_2_ production

The evaluation of H_2_ production is similar with the widely-used photocatalytic water splitting evaluation. It was carried out in a closed gas circulation and evacuation system using a 450 W high-pressure Hg lamp (Ushio-UM452). 500 mL CH_3_OH-H_2_O (10% CH_3_OH, 90% H_2_O) was used as reaction reagent. Semiconductor-grade pure CH_3_OH (>99.99%) and pure H_2_O (18 MΩ) was used for experiment, which was obtained from a Milli-Q water purification system. Before irradiation, the reaction system was thoroughly degassed by evacuation in order to drive off the air inside. The amount of evolved H_2_ and O_2_ was determined by an on-line gas chromatograph (Agilent, GC-7890, TCD, Ar carrier). Analytically pure SiO_2_ and Al_2_O_3_ particles were purchased from Tianjin Kemiou Chemical Reagent Co., 5.0 g samples were grinded by ball-milling for 12 hours for reaction, 0.05 wt% Pt was deposited by *in-situ* photo-deposition method at the initial stage of the reaction.

### Characterization

The emission spectrum of Hg lamp was characterized by a commercial spectral radiometer, AvaSolar (Serialnr: S1101239U1, Grating: UA, 200–1100 nm. Option: Slit-50, OSC-UA. Software: AvaSolar Avasoft-full irrad.). The absorption of different solutions was collected on a UV-vis spectrophotometer (JASCO V-650). The scanning rate is 100 nm/min, the scanning range is from 200 to 600 nm. Photoluminescence spectra were carried out on a FLS920 fluorescence spectrometer (Edinburgh Instruments). The laser at 266 nm comes from the double-frequency of a DPSS 532 Model 200 532 nm laser and the laser line at 325 nm of a He-Cd laser was used as exciting sources, respectively. Electron paramagnetic resonance (EPR) was recorded on a Brucker EPR A200 spectrometer. The settings for the EPR spectrometer were as follows: center field, 3486.70 G; sweep width, 100 G; microwave frequency, 9.82 GHz; modulation frequency, 200 kHz; power, 20.00 mW. Magnetic parameters of the radicals detected were obtained from direct measurements of magnetic field and microwave frequency.

## Additional Information

**How to cite this article**: Li, R. *et al.* Photo-induced H_2_ production from a CH_3_OH-H_2_O solution at insulator surface. *Sci. Rep.* **5**, 13475; doi: 10.1038/srep13475 (2015).

## Supplementary Material

Supplementary Information

## Figures and Tables

**Table 1:** Photo-induced H_2_ production from a CH_3_OH-H_2_O solution under light irradiation at different ranges.

Entry	Wavelength (nm)	The amount of H_2_ evolution (μmol/h)
1	Full spectrum	83.1
2	>400	0.2
3	>340	2.3
4	>300	7.6
5	>240	82.5

Reaction conditions: 500 mL CH_3_OH-H_2_O solution (10% CH_3_OH), 450 W Hg lamp was used as light source, inner irradiation type. The light range was controlled with different light-absorbing solution.

## References

[b1] Maeda K. *et al.* Photocatalyst releasing hydrogen from water. Nature 440, 295–295 (2006). 16541063 10.1038/440295a

[b2] Ni M., Leung M. K., Leung D. Y. & Sumathy K. A review and recent developments in photocatalytic water-splitting using TiO_2_ for hydrogen production. Renew. Sust. Energ. Rev. 11, 401–425 (2007).

[b3] Kudo A. & Miseki Y. Heterogeneous photocatalyst materials for water splitting. Chem. Soc. Rev. 38, 253–278 (2009). 19088977 10.1039/b800489g

[b4] Chen X., Shen S., Guo L. & Mao S. S. Semiconductor-based photocatalytic hydrogen generation. Chem. Rev. 110, 6503–6570 (2010). 21062099 10.1021/cr1001645

[b5] Li R. *et al.* Spatial Separation of Photogenerated Electrons and Holes among {010} and {110} Crystal Facets of BiVO_4_. Nat. Commun. 4, 1432, 10.1038/ncomms2401 (2013). 23385577

[b6] Papaconstantinou E. Photochemistry of polyoxometallates of molybdenum and tungsten and/or vanadium. Chem. Soc. Rev. 18, 1–31 (1989).

[b7] Ishikawa M. *et al.* Photolysis of organopolysilanes. Photochemical behavior of phenylethynyldisilanes. J. Am. Chem. Soc. 104, 2872–2878 (1982).

[b8] Dougherty D. A. Spin control in organic molecules. Acc. Chem. Res. 24, 88–94 (1991).

[b9] Shawali A. S. Reactions of heterocyclic compounds with nitrilimines and their precursors. Chem. Rev. 93, 2731–2777 (1993).

[b10] Yi H. *et al.* Photocatalytic H_2_ production from methanol aqueous solution over titania nanoparticles with mesostructures. Int. J. Hydrogen. Energy 33, 672–678 (2008).

[b11] Moon S.-C., Matsumura Y., Kitano M., Matsuoka M. & Anpo M. Hydrogen production using semiconducting oxide photocatalysts. Res. Chem. Intermediates 29, 233–256 (2003).

[b12] Ikeda S. *et al.* Mechano-catalytic overall water splitting. Chem. Commun. 20, 2185–2186 (1998).

[b13] Domen K., Kondo J. N., Hara M. & Takata T. Photo-and mechano-catalytic overall water splitting reactions to form hydrogen and oxygen on heterogeneous catalysts. Bull. Chem. Soc. Jp. 73, 1307–1331 (2000).

[b14] Hara M. *et al.* Mechano-catalytic overall water splitting (II) nafion-deposited Cu_2_O. Appl. Catal. A: General 190, 35–42 (2000).

[b15] Takata T. *et al.* Mechano-catalytic overall water splitting on some oxides (II). Appl. Catal. A: General 200, 255–262 (2000).

[b16] Domen K. *et al.* Mechano-catalytic overall water-splitting into hydrogen and oxygen on some metal oxides. Appl. Energy 67, 159–179 (2000).

[b17] Hitoki G. *et al.* Mechano-catalytic overall water splitting on some mixed oxides. Catal. Today 63, 175–181 (2000).

[b18] Kondo J. Mechano-catalysis-a novel method for overall water splitting. Phys. Chem. Chem. Phys. 1, 4485–4491 (1999).

[b19] Hara M. *et al.* A study of mechano-catalysts for overall water splitting. J. Phys. Chem. B 104, 780–785 (2000).

[b20] Chamberlain G. & Simons J. Polarised photofluorescence excitation spectroscopy: A new technique for the study of molecular photodissociation. Photolysis of H_2_O in the vacuum ultraviolet. Chem. Phys. Lett. 32, 355–358 (1975).

[b21] Schultz M., Heitlinger M., Mihelcic D. & Volz-Thomas A. A. Calibration source for peroxy radicals with built-in actinometry using H_2_O and O_2_ photolysis at 185 nm. J. Geophys. Res. 100, 18811–18816 (1995).

[b22] Buenker R., Olbrich G., Schuchmann H., Schürmann B. & Von Sonntag C. Photolysis of methanol at 185 nm. Quantum-mechanical calculations and product study. J. Am. Chem. Soc. 106, 4362–4368 (1984).

[b23] Jacox M. E. & Milligan D. E. Matrix isolation study of the vacuum-ultraviolet photolysis of methanol: The infrared spectrum of the CH_2_OH free radical. J. Mol. Spectrosc. 47, 148–162 (1973).

[b24] Salahub D. & Sandorfy C. The far-ultraviolet spectra of some simple alcohols and fluoroalcohols. Chem. Phys. Lett. 8, 71–74 (1971).

[b25] Nee J., Suto M. & Lee L. Photoexcitation processes of CH_3_OH: Rydberg states and photofragment fluorescence. Chem. Phys. 98, 147–155 (1985).

[b26] Burton G. R., Chan W. F., Cooper G. & Biron C. Absolute oscillator strengths for photoabsorption (6–360 eV) and ionic photofragmentation (10–80 eV) of methanol. Chem. Phys. 167, 349–367 (1992).

[b27] Thomas J. K. Physical aspects of radiation-induced processes on SiO_2_, γ-Al_2_O_3_, zeolites, and clays. Chem. Rev., 105 (5), 1683–1734 (2005). 15884787 10.1021/cr020378a

[b28] Du C. *et al.* Observation and alteration of surface states of hematite photoelectrodes. J. Phys. Chem. C, 118, 17054–17059 (2014).

[b29] Mashkovtsev R. I. & Pan Y. Stable states of E” defects in alpha-quartz. Eur. Phys. Lett., 98, 56005 (2012).

[b30] Mashkovtsev R. I. & Pan Y. Triplet states of oxygen-vacancy defects in a-quartz: Center E“9. Eur. Phys. Lett., 107, 36005 (2014).

[b31] Dzyuba V., Kulchin Y. & Milichko V. Nanocomposites-new trends and developments (eds Ebrahimi Farzad) Ch.15, 393–420 (InTech, 2012).

[b32] Wu M., Alivov Y. & Morkoç H. High-κ dielectrics and advanced channel concepts for Si MOSFET. J. Mater. Sci.-Mater. El. 19, 915–951 (2008).

[b33] Xu Y. & Schoonen M. A. A. The absolute energy positions of conduction and valence bands of selected semiconducting minerals. Am. Mineral. 85, 543–556 (2000).

[b34] Furukawa S., Ohno Y., Shishido T., Teramura K., Tanaka T. Reaction mechanism of selective photooxidation of amines over niobium oxide: visible-light-induced electron transfer between adsorbed amine and Nb_2_O_5_. J. Phys. Chem. C 117, 442–450 (2013).

[b35] Furukawa S., Ohno Y., Shishido T., Teramura K., Tanaka T. Selective amine oxidation using Nb_2_O_5_ photocatalyst and O_2_. ACS Catal. 1, 1150–1153 (2011).

[b36] Furukawa S. *et al.* Mechanism of photooxidation of alcohol over Nb_2_O_5_. J. Phys. Chem. C 113, 18713–18718 (2009).

