# Supplementary material for: Photo-induced H2 production from a CH3OH-H2O solution at insulator surface
Source: Sci Rep. 2015 Aug 28;5:13475. doi: 10.1038/srep13475 (PMC4551957; doi:10.1038/srep13475)
Supplement: Supplementary Information [file srep13475-s1.pdf]

Supporting Information

Photo-induced H_2 production from a CH_3OH-H_2O solution at insulator surface

Rengui Li[1], Xiuli Wang[1], Shaoqing Jin[1,2], Xin Zhou[1], Zhaochi Feng[1], Zheng Li[1,2], Jingying Shi[1], Qiao Zhang[1] and Can Li[1*]

1. *State Key Laboratory of Catalysis, Dalian Institute of Chemical Physics, Chinese Academy of Sciences, Dalian National Laboratory for Clean Energy, Zhongshan Road 457, Dalian, 116023, China.*
2. *University of Chinese Academy of Sciences, Beijing, 100049, China.*

[*] *Corresponding author: Prof. Can Li, canli@dicp.ac.cn*

Performance evaluation.

The performance evaluation method is similar with the widely used photocatalytic water splitting evaluation. It was carried out in a closed gas circulation and evacuation system using a 450W high-pressure Hg lamp (Ushio-UM452). 500 mL CH_3OH-H_2O (10% CH_3OH, 90% H_2O) was used as reaction reagent. Semiconductor-grade pure CH_3OH (>99.99%) and pure H_2O (18 MΩ) was used for experiment, which was obtained from a Milli-Q water purification system. Before irradiation, the reaction system was thoroughly degassed by evacuation in order to drive off the air inside. The amount of evolved H_2 and O_2 was determined by an on-line gas chromatograph (Agilent, GC-7890, TCD, Ar carrier).

To rational control the emission range of light source, we chose several kinds light-absorbing solutions (5.0 mol/L $NaNO_2$, $NaNO_3$ and Na_2CO_3) to be filled in the filter layer. The above solutions were used to absorb the different range of light so that the specific range of light reach the reaction solution.

Analytically pure SiO_2 and Al_2O_3 particles were purchased from Tianjin Kemiou Chemical Reagent Co., 5.0 g samples were grinded by ball-milling for 12 hours for reaction in Figure 4, 0.05wt% Pt was deposited by in-situ photo-deposition method at the initial stage of the reaction.

Characterization.

The emission spectrum of Hg lamp was characterized by a commercial spectral radiometer, AvaSolar (Serialnr: S1101239U1, Grating: UA, 200-1100 nm. Option: Slit-50, OSC-UA. Software: AvaSolar Avasoft-full irrad.). The absorption of different

solutions was collected on a UV-vis spectrophotometer (JASCO V-650). The scanning rate was 100 nm/min, the scanning range is between 200 and 600 nm. Photoluminescence spectra were carried out on a FLS920 fluorescence spectrometer (Edinburgh Instruments). The laser at 266 nm comes from the double-frequency of a DPSS 532 Model 200 532 nm laser and the laser line at 325 nm of a He-Cd laser was used as exciting sources, respectively. The settings for the EPR spectrometer were as follows: center field, 3486.70 G; sweep width, 100 G; microwave frequency, 9.82 GHz; modulation frequency, 200 kHz; power, 20.00 mW. Magnetic parameters of the radicals detected were obtained from direct measurements of magnetic field and microwave frequency. For EPR experiment, quartz sand particles were treated with different solutions ($FeCl_3$, $CdCl_2$, $CHCl_3$ and $CuCl_2$) for 5 h, and then centrifuged, washed by water and dried at room temperature overnight for EPR characterization. The concentrations of the treatment solutions were 1.0 mM for metal ions and 1% for $CHCl_3$ solution.

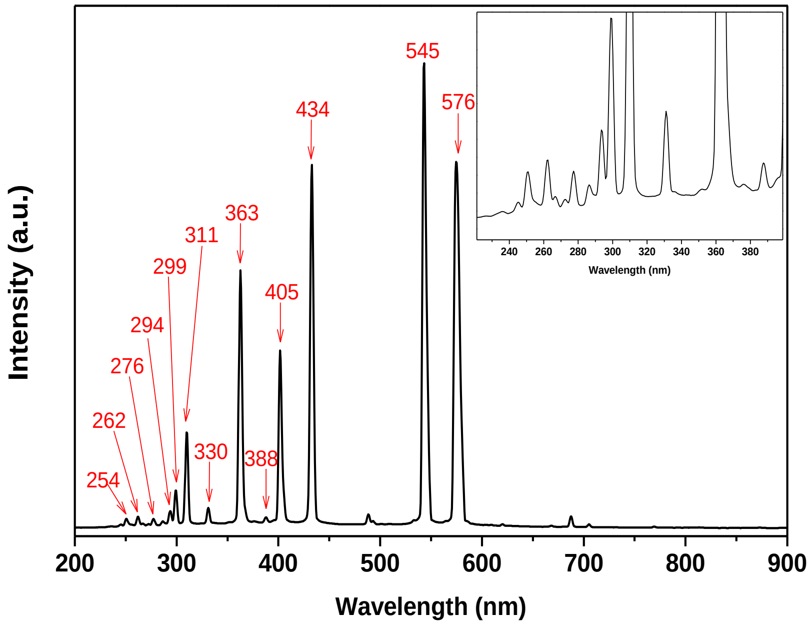

Figure S1. The emission spectrum of Hg lamp used in the experiment. Inset shows the magnification of the spectrum in the UV range. The spectrum was collected by spectral radiometer (AvaSolar).

The emission spectrum of Hg lamp source shows five strong peaks located at 363 nm, 405 nm, 434 nm, 545 nm and 576 nm, together with a series of weak peaks range from 240 nm to 311 nm. This spectrum is the characteristic of a standard Hg lamp from commercial sources.

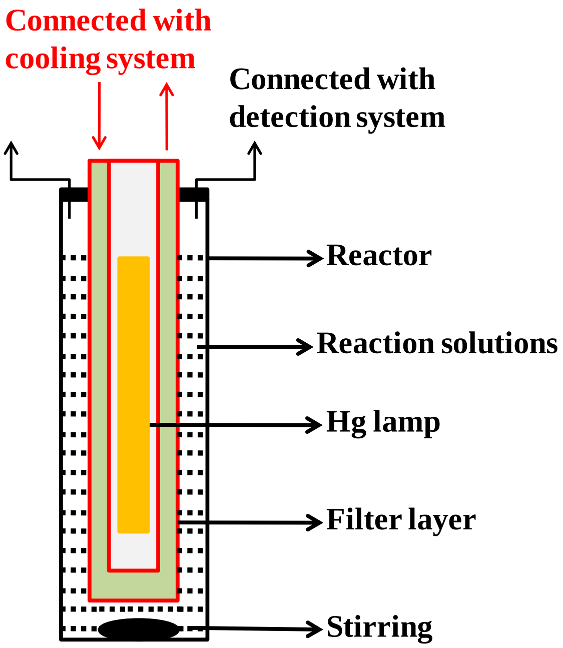

Figure S2. The scheme for the structure of reactor used in the experiment.

The reactor is composed of four parts, reactor, light filter layer, cooling system and light source. The produced gas is filled in a closed system and connected with GC for further detection. It should be pointed out that the filter layer are made of quartz which can only absorb the light with wavelength shorter than 200 nm, so it will not affect the light irradiation of the Hg lamp. The Pyrex-made reactor was used in the experiment. To accurately tune the required range of light spectrum irradiated to the reaction solution, different light-absorbing solutions which can absorb specific range of light were introduced in the filter layer to make sure the required range of light reach to the reaction solution. The solutions are also filled in circulating pump to keep the temperature at 288 K (cooling system).

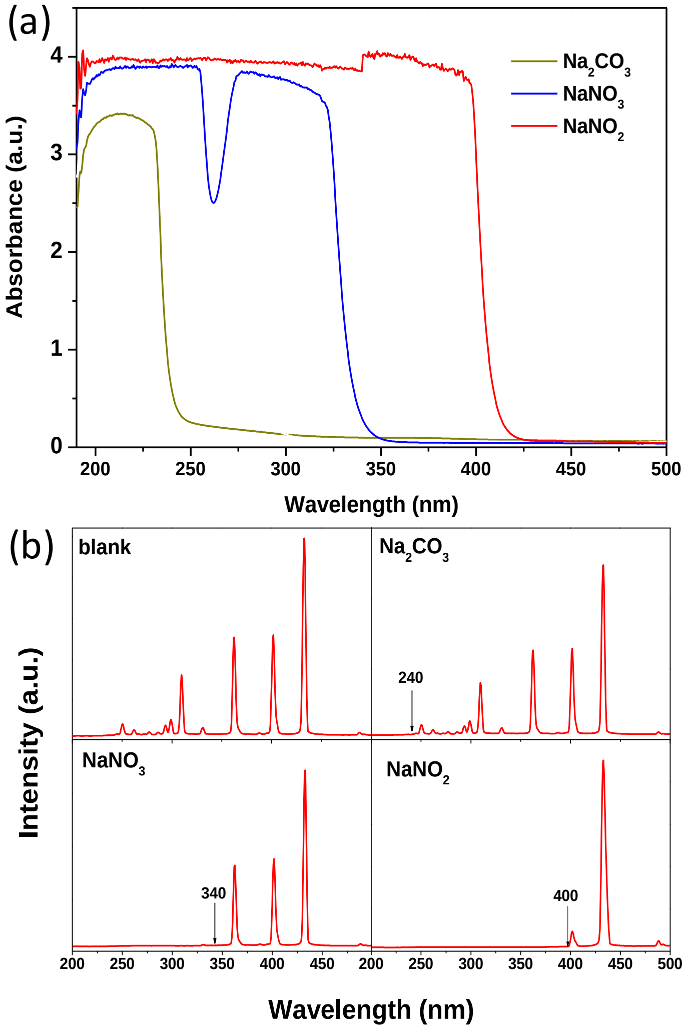

Figure S3. (a) The absorption spectrum of different chosen solutions which were introduced in the filter layer to tune the required light range. (b) The spectrum of the Hg lamp after the filter layer filled with chosen solutions.

To determine which range of light source plays a role in photo-induced H_2 production in CH_3OH-H_2O solution, we chose several types of light-absorbing solutions (Na_2CO_3, $NaNO_3$ and $NaNO_2$) to filter the light at the absorption edges approximately at 240 nm, 340 nm, and 400 nm. We further detected the spectra of the Hg lamp with the chosen solutions in the filter layer to confirm the required light ranges. It indicates that all of them are in good agreement with their absorption spectra, so we can select the specific range of light for reaction.

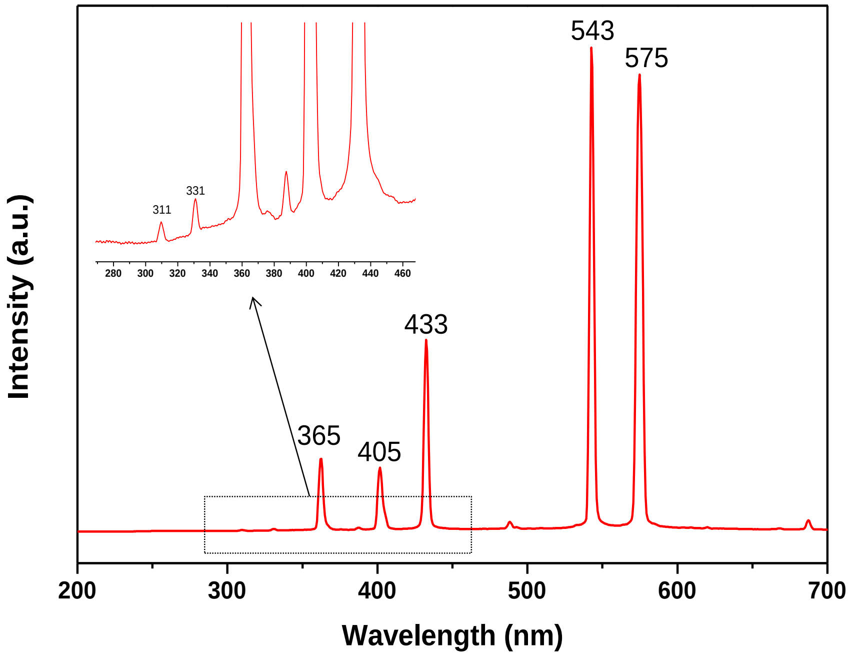

Figure S4.The spectrum of the Hg lamp irradiated on the reaction solution when the filter layer is made of glass. Glass can absorb the light ranged in the deep ultraviolent, so when the glass filter layer is used, the light with wavelength less than 300 nm was all absorbed by glass.

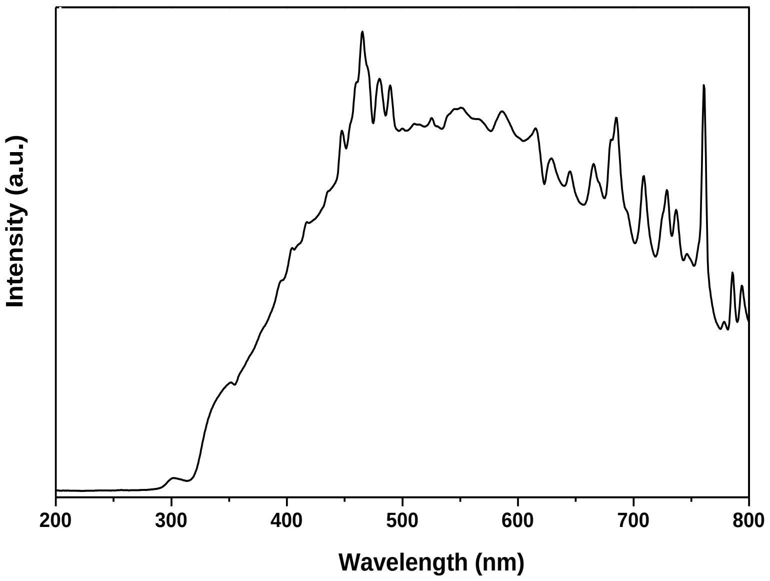

Figure S5. The spectrum of the Xe lamp. The spectrum was collected by spectral radiometer (AvaSolar).

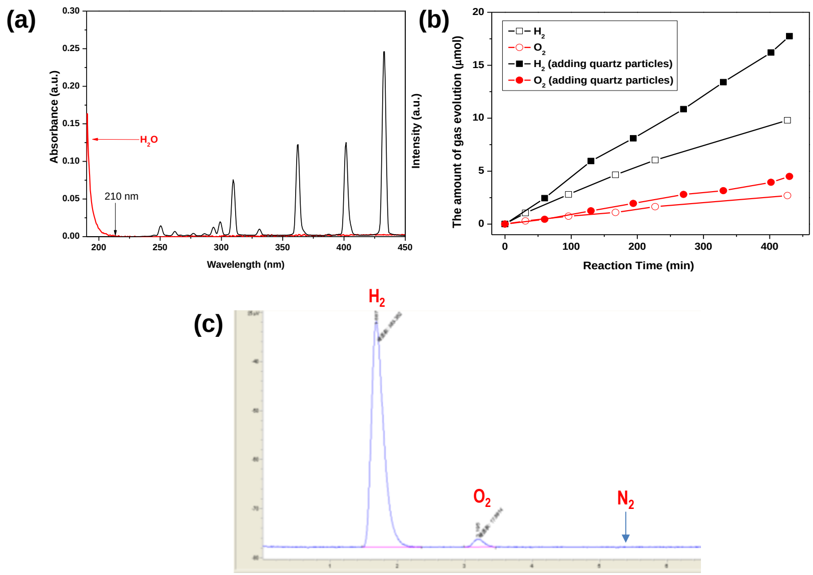

Figure S6. (a) The absorption spectrum of pure H_2O and the emission spectrum of Hg lamp; (b) Photo-induced H_2 and O_2 production under the irradiation of Hg lamp; (c) The detection of H_2 and O_2 from gas chromatography (GC).

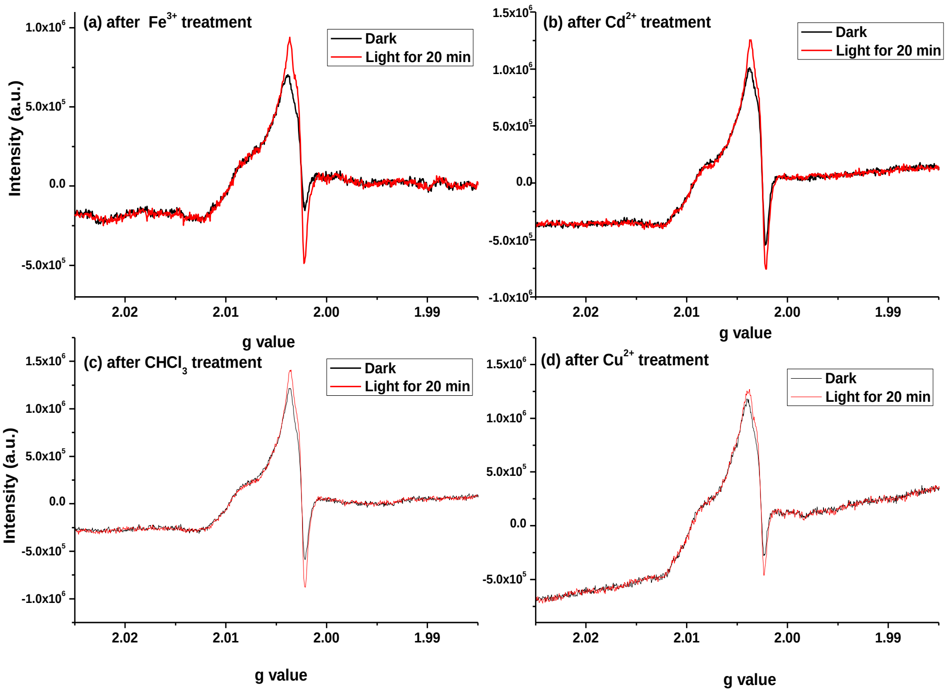

Figure S7. EPR spectra of the quartz sand particles treated by different electron scavengers with or without UV light irradiation. (a) Fe^{3+}, (b) Cd^{2+}, (c) $CHCl_3$ and (d) Cu^{2+}. Conditions: quartz particles were treated with different solutions ($FeCl_3$, $CdCl_2$, $CHCl_3$ and $CuCl_2$), washed by water, dried at room temperature overnight, and then irradiated by 100 W Hg lamp for 20 min. EPR signals were collected under the light irradiation.

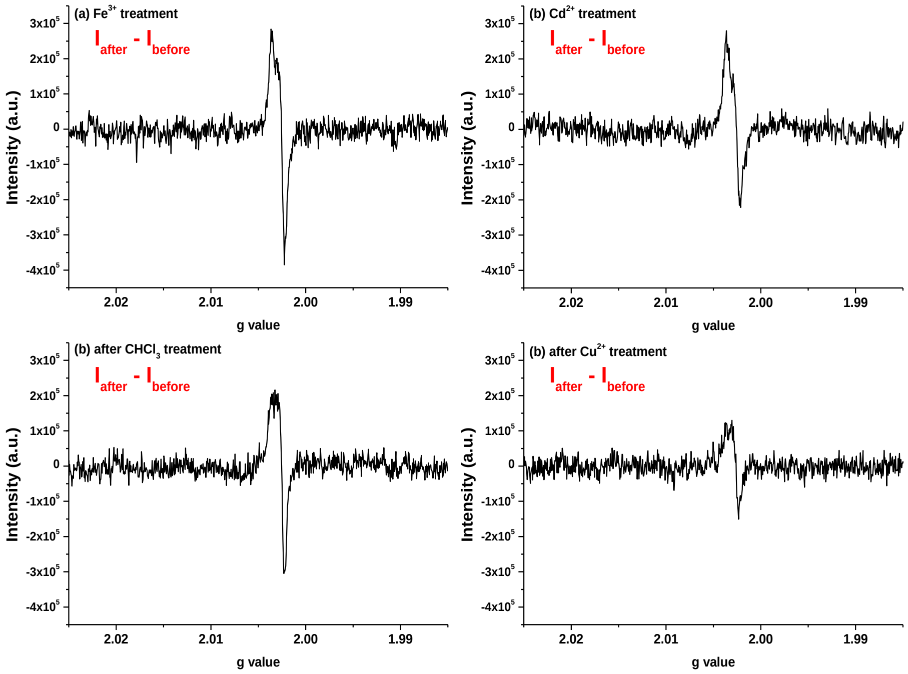

Figure S8. The differential of EPR spectra before and after UV light irradiation (I_{after} and I_{before} are obtained by from Figure S6).

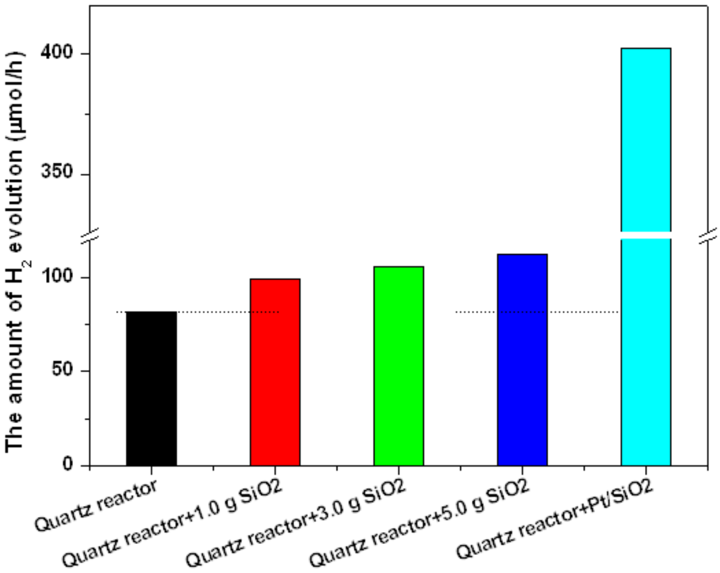

Figure S9. The optimization of different adding amount of quartz sand particles for photo-induced H_2 production from a CH_3OH-H_2O solution.

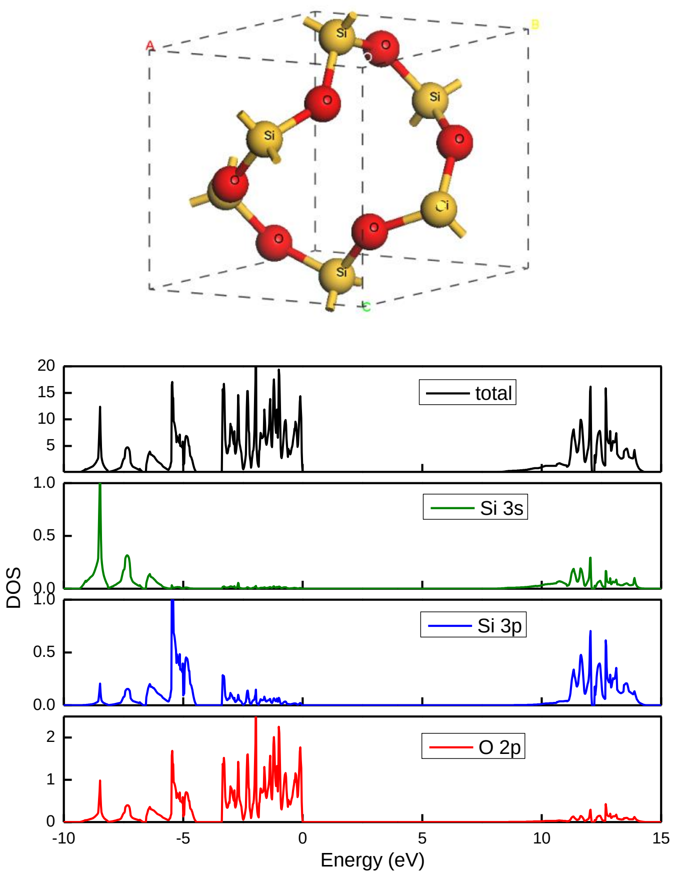

Figure S10. Density of states (DOS) of SiO_2.

All the DFT calculations were performed with the VASP (Vienna Ab initio Simulation Package) code.[1,2] Full optimization of cell parameters of quartz has been carried out by the Perdew-Burke-Ernzerhof functional within the generalized gradient approximation.[3-5] The cutoff energy of 400 eV and 7×7×7 Monkhorst-Pack type *k*-point sampling have been adopted. The calculated lattice parameters, a=b=4.851 Å, c=5.361Å, and γ=120°, are in good agreement with experimental data.[6] Total and projected density of states have been examined by the Heyd-Scuseria-Ernzerhof hybrid functional (HSE06).[7-9] The calculated band gap is about 8.0 eV and the valance band of SiO_2 was mainly contributed by O 2p orbital.

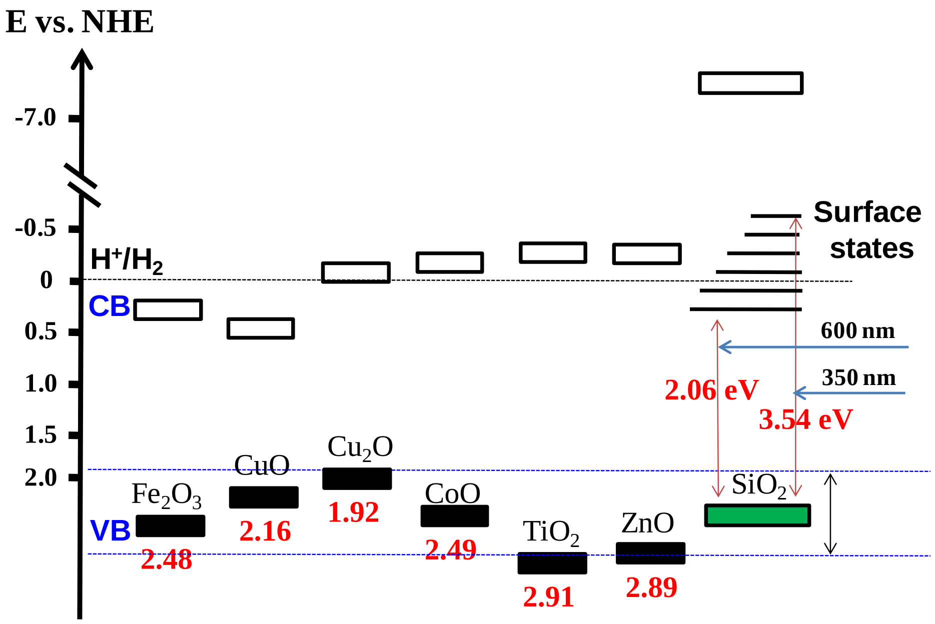

Figure S11. The estimation for the energy structure of quartz.

The absolute positions of valence band for all these oxides are collected. The absolute positions of valence band for all these oxides are collected. The valence band of these oxides are mainly contributed by O 2p orbital, so it can be speculated that the valence band of SiO_2 is located at comparable position with them.

Table S1. The photolysis of other kinds of sacrificial reagents that has been used in photocatalytic H_2 production.

**Entry**	**Organic reagents**	**H_2 production (μmol/h)**
1	methanol	83.1
2	ethanol	181.1
3	isopropyl alcohol	195.4
4	lactic acid	96.8

Reaction conditions: 500 mL organic reagent-H_2O solution (10% organic reagent), 450 W Hg lamp was used as light source, inner irradiation type. 18 MΩ H_2O was used for experiment, which was obtained from a Milli-Q water purification system.

Table S2. The influence of electron scavengers to the photo-induced H_2 production in CH_3OH-H_2O solution.

Entry	Adding electron scavengers	The amount of H_2 evolution (μmol/h)
1	blank	83.1
2	Cd^{2+}	26.0
3	$CHCl_3$	30.7

As Fe^{3+} and Cu^{2+} have strong absorption in the light range, both of them were not used in this experiment to exclude the possible photochemical process.

Reaction condition: 500 mL CH_3OH-H_2O solution (10% CH_3OH), 450 W high-pressure Hg lamp. The concentration of Cd^{2+} was 1.0 mmol/L and the concentration of $CHCl_3$ was 1% in volume.

.

Table S3. Photo-induced H_2 production performance under the irradiation of lasers.

**Wavelength**	**H_2 production (μmol/h/W)**	
	**Quartz-window**	**Pyrex-window**
266 nm	2.8	<0.1
355 nm	0.65	0.15

Reaction condition: 100 mL CH_3OH-H_2O solution (50% CH_3OH), irradiation time: 2 h. The system was first vacuumed and saturated with Ar, and then irradiated with 266 nm and 355 nm lasers.

Table S4. Photo-induced H_2 production performance with or without P25

**Entry**	**Photocatalyst**	**H_2 production (μmol/h)**
1	P25	81.9
2	Pt/P25	2795.3
3	blank	83.1

Reaction conditions: with or without adding of Degussa P25, 0.10 g, 0.1wt% Pt as cocatalyst, in-situ photo-deposition. 500 mL CH_3OH-H_2O solution (10% CH_3OH), 450 W Hg lamp was used as light source, inner irradiation type. 18 MΩ H_2O was used for experiment, which was obtained from a Milli-Q water purification system.

References.

(1) G. Kesse, J. Furthmüller, Efficient iterative schemes for ab initio total-energy calculations using a plane-wave basis set. *Phys. Rev. B* **54**, 11169-11186 (1996).

(2) G. Kesse, J. Furthmüller, Efficiency of *ab-initio* total energy calculations for metals and semiconductors using a plane-wave basis set. *Comput. Mater. Sci.* **6**, 15-50 (1996).

(3) J. P. Predew, K. Burke, M. Ernzerhof, Generalized gradient approximation made simple. *Phys. Rev. Lett.* **77**, 3865-3868 (1996).

(4) P. Blöchk, Projector augmented-wave method. *Phys. Rev. B* **50**, 17953-17979 (1994).

(5) G. Kresse, D. Joubert, From ultrasoft pseudopotentials to the projector augmented-wave method. *Phys. Rev. B* **59**, 1758-1775 (1999).

(6) Levien, L.; Prewitt, C. T.; Weidner, D. J. Structure and elastic properties of quartz at pressure. *Am. Mineral.* **65**, 920-930 (1980).

(7) Heyd, J.; Scuseria, G. E.; Ernzerhof, M. Hybrid functionals based on a screened Coulomb potential. *J. Chem. Phys.* **118**, 8207 (2003).

(8) Heyd, J.; Scuseria, G. E.; Ernzerhof, M. Hybrid functionals based on a screened Coulomb potential. *J. Chem. Phys.* **124**, 219906 (2006).

(9) Paier, J.; Marsman, M.; Hummer, K.; Kress, G.; Gerber, I. C.; Angyan, J. G. Screened hybrid density functionals applied to solids. *J. Chem. Phys.* **125**, 249901 (2006).
